# First Degree Relatives of Patients with Celiac Disease Harbour an Intestinal Transcriptomic Signature that Might Protect them from Enterocyte Damage

**DOI:** 10.1038/s41424-018-0059-7

**Published:** 2018-10-08

**Authors:** Pragyan Acharya, Rintu Kutum, Rajesh Pandey, Asha Mishra, Rohini Saha, Akshay Munjal, Vineet Ahuja, Mitali Mukerji, Govind K. Makharia

**Affiliations:** 1 Department of Biochemistry, All India Institute of Medical Sciences, New Delhi, India; 2 Genomics and Molecular Medicine and CSIR-TRISUTRA Ayurgenomics Unit, Council for Scientific and Industrial Research-Institute of Genomics and Integrative Biology, New Delhi, India; 3 Academy of Scientific and Innovative Research, CSIR-IGIB, Delhi, India; 4 Department of Gastroenterology and Human Nutrition, All India Institute of Medical Sciences, New Delhi, 110029 India; 5 Present Address: Mammalian Genetics Unit, MRC Harwell Institute, Harwell Science and Innovation Campus, Oxfordshire, OX11 0RD United Kingdom

## Abstract

**Introduction:**

Celiac disease (CeD) is an autoimmune enteropathy which affects approximately 0.7% of the global population. While first-degree relatives (FDR) of patients with CeD have a 7.5% risk of developing enteropathy, many remain protected. Therefore, intestinal mucosa of FDR might have protective compensatory mechanisms against immunological injury. We have explored the protective mechanisms that may be active in intestinal mucosa of FDR.

**Methods:**

Intestinal mucosal biopsies (4–5 pieces) from treatment naïve patients with CeD (*n* = 12), FDR (*n* = 12) (anti-tTG negative) and controls (*n* = 12) (anti-tTG negative) were obtained from each individual and subjected to microarray analysis using HT-12-v4 Human Expression BeadChips (Illumina). Differential gene expression analysis was carried out among CeD, FDR and controls; and resulting gene lists were analyzed using gene ontology and pathway enrichment tools.

**Results:**

Patients with CeD, FDR and control groups displayed significant differential gene expression. Thirty seven genes were upregulated and 372 were downregulated in the intestinal mucosa of FDR in comparison to CeD and controls. Pseudogenes constituted about 18% (315/1751) of FDR differentially expressed genes, and formed “clusters” that associated uniquely with individual study groups. The three study groups segregated into distinct clusters in unsupervised (PCA) and supervised (random forests) modelling approaches. Pathways analysis revealed an emphasis on crypt-villous maintenance and immune regulation in the intestinal mucosa of FDR.

**Conclusions:**

Our analysis suggests that the intestinal mucosa of celiac FDR consist of a unique molecular phenotype that is distinct from CeD and controls. The transcriptomic landscape of FDR promotes maintenance of crypt-villous axis and modulation of immune mechanisms. These differences clearly demonstrate the existence of compensatory protective mechanisms in the FDR intestinal mucosa.

## Introduction

Celiac disease (CeD) is an autoimmune enteropathy, which results from an interaction between genetic and environmental factors. The environmental factor in CeD is gluten, a storage protein present in wheat, rye and barley^1^. While about 40% of genetic predisposition in CeD can be explained by the HLA-DQ2/DQ8 haplotype, many more genes have now been found to be associated with CeD and possibly more are awaiting discovery^2^.

CeD is a global disease and a recent systematic review and meta-analysis shows that the global prevalence of CeD is 0.7% (95% CI, 0.5%–0.9%)^3^. Furthermore, first-degree relatives (FDR) of patients with CeD are at a higher risk of developing CeD. Overall, approximately 7.5% of FDR develop CeD^4^. While about one-third of the global population has HLA-DQ2/DQ8 haplotype, approximately 80–85% of FDR and almost 90% of patients with CeD have HLA-DQ2/DQ8 haplotype^5^. Therefore, the higher risk of developing gluten-induced enteropathy in FDR is well explained by their higher probability of harbouring the HLA DQ2/DQ8 alleles than the general population. While almost 80% of FDR are genetically susceptible to develop CeD, only a proportion of them do so. This suggests the presence of protective mechanisms in the intestinal mucosa of the FDR.

In the present study, we have explored the hypothesis that protective mechanisms exist in the intestinal mucosa of FDR of patients with CeD. Towards this, we have carried out global transcriptomics analysis through microarray of intestinal mucosal biopsies obtained from FDR, patients with CeD and controls. We find features indicative of processes that might have protective roles in the intestinal mucosa of celiac FDR. This is the first study that analyzes the transcriptomic differences in the intestinal mucosal biopsies of FDR, giving us a glimpse into mechanisms that might protect genetically predisposed individuals from gluten-induced enteropathy.

## Methods

This study was conducted at All India Institute of Medical Sciences (AIIMS), New Delhi and CSIR-Institute of Genomics and Integrative Biology (CSIR-IGIB), New Delhi.

### Study groups

Three groups of participants were included in this study including treatment naïve patients with CeD, unpaired FDR who consented to be a part of this study, and the controls. Ethical clearance was obtained from the institute ethics committee of AIIMS New Delhi (Ref. No. IEC/NP-220/2010).

#### (A) Treatment naïve patients with CeD (*n* = 12)

Twelve newly diagnosed patients having definitive CeD were included in this study group. All of them had high (ten folds of normal) levels of anti-tissue transglutaminase antibody (anti-tTG Ab), advanced villous abnormalities (modified Marsh 3c) on histological assessment, and HLA-DQ2 haplotype. Patients with history of active non-steroidal anti-inflammatory drugs (NSAID) or proton pump inhibitors use, those who had received antibiotics within preceding eight weeks, those with intestinal resection, and achlorhydria, as well as pregnant and lactating mothers were excluded from this study. Those selected were put on gluten-free diet under the care of a nutritionist.

#### (B) FDR (*n* = 12)

As a part of routine screening, asymptomatic FDR who were anti-tTG Ab negative were invited to participate in this study. They were tested for HLA-DQ2/DQ8 and underwent endoscopic examination to obtain duodenal biopsies. The biopsies were assessed for villous abnormalities using modified Marsh grading. Finally, asymptomatic anti-tTG Ab negative FDR with HLA-DQ2 haplotype and with evidence of no enteropathy were included in this study.

#### (C) Controls (*n* = 12)

Patients with functional dyspepsia undergoing endoscopic examination for their diagnosis were included as controls. They were screened for CeD using anti-tTG Ab. All of them had a normal esophagus, stomach and duodenum on endoscopic examination, had normal crypt villous ratio and were anti-tTG Ab negative. They were also assessed for HLA haplotype. Six of them had HLA-DQ2/DQ8 haplotype and none were FDR of diagnosed patients with celiac disease.

### Procedures and data analysis

#### (A) Biopsy sampling and RNA extraction

All the above participants underwent upper GI endoscopic examination, as described earlier. Multiple mucosal biopsies were obtained using spike biopsy forceps. Four bits of tissues in 10% formal saline were sent for histological examination for villous architecture assessment. Other 4–6 bits of mucosal biopsies were immediately transferred in RNAlater for gene expression analysis. RNA extraction was carried out using RNeasy mini kit (Qiagen). RNA quantity and quality were estimated using RNA 6000 Nano kit on 2100 Agilent Bioanalyzer (Agilent technologies, Palo Alto, California, USA). The yield of RNA varied from 12 to 20 µg of total RNA using 25 mg of tissue.

#### (B) Microarray experiment

An Illumina TotalPrep RNA Amplification Kit was used to prepare the samples for hybridization on Illumina BeadChips. The integrity of RNA was checked using Bioanalyzer (Agilent 2100). The protocol included first-strand cDNA synthesis from 500 ng of total RNA using reverse transcriptase, followed by simultaneous second strand cDNA synthesis with DNA polymerase. This process also degraded the residual RNA with RNase H. In vitro transcription of the purified cDNA was carried out by T7 RNA polymerase using biotinylated primers. The purified cRNA concentration was estimated using NanoDrop spectrophotometer and then hybridized to HT-12-v4 Human Expression BeadChips (Illumina, San Diego, CA, USA) following the manufacturer’s instructions. Labelled cRNA was detected by hybridization to 50-mer probes on the BeadChip. Subsequent steps included stringent washing, blocking and staining with streptavadin-Cy3. Scanning was done using Illumina iScan. Fluorescence emission by Cy3 was quantitatively detected for downstream analysis. Experiments were carried out using samples obtained from treatment naïve patients with CeD, FDR, and controls.

#### (C) qPCR validation

Microarray data were validated by qPCR of selected genes in independently recruited participants in the three study groups CeD, FDR and controls. Briefly, new participants were recruited into the three study groups and duodenal biopsies were obtained from them as described above. RNA was prepared from the biopsies followed by cDNA preparation from about 2 μg of RNA from all samples, keeping the input RNA constant. This was followed by qPCR of selected target genes using human 18S rRNA as a control. SYBR Green chemistry and Bio-Rad CFX-96 well plate real-time PCR machine were used. Details of the procedure and primer sequences are included in Supplementary Methods as per MiQE guidelines.

#### (D) Differential gene expression analysis

Raw intensity signal without background correction and imputation was extracted using Illumina GenomeStudio software. Background correction was performed using lumi^6^ package in R. In order to improve stringency of probe selection, we have only included probes with detection *p*-value ≤ 0.05 observed in at least six out of 12 samples per array. Probe-wise normality test was performed and parametric and non-parametric tests were carried out accordingly. One-way ANOVA test and Kruskal-Wallis rank sum test were performed to identify probes that exhibit significant differences between any of the groups. This was followed by pair wise comparisons using *t-*test and Mann-Whitney test to identify probes that were differentially expressed. All the subsequent analyses were carried out on gene sets that showed differential gene expression with a Bonferroni corrected *p* value ≤ 0.05. Gene Ontology and pathway enrichment analysis were carried out with clusterProfiler^7^ and MsigDB^8^ on gene sets that fulfilled an additional criteria of log_2_(fold change) ≤ −1 corresponding to a fold-change of 0.5 (as down-regulated) or log_2_(fold-change) >  1 corresponding to fold-change of 2 (as up-regulated)^9^. An additional pathways analysis using Reactome^10^, was carried out on all significant differentially expressed genes without a log_2_(fold-change) restriction.

#### (E) Gene expression pattern analysis among CeD, FDR and Controls

To understand the overall pattern of gene expression for a specific gene in the three study groups, mean expression values of a given significant probe (gene) was calculated for each group (mean expression values were represented as *µ*_CeD_, *µ*_FDR_ and *µ*_control_). Then the probes were segregated into four distinct groups namely, *µ*_(FDR)_ < *µ*_(CeD)_ < *µ*_(Control)_, *µ*_(CeD)_ < *µ*_(Control)_ < *µ*_(FDR)_, *µ*_(FDR)_ < *µ*_(Control)_ < *µ*_(CeD)_, and *µ*_(CeD)_ < *µ*_(FDR)_ < *µ*_(Control)_. For each group, gene ontology analysis was performed using clusterProfiler.

#### (F) Unsupervised and supervised analysis of differentially expressed probes

Principal component analysis was carried out to infer whether the differentially expressed genes (1751 probes x 36 samples) could form natural clusters in an unsupervised manner. Further, supervised analysis using random forests^11^ modelling approach was also performed to identify minimal set of probes that could stratify the three study groups. To identify the minimal set, variable selection using Boruta^12^ algorithm was performed. To reduce the computational burden, probes were binned into size of 100 and for each bin, Boruta algorithm was performed with threshold of *p*-value ≤ 0.001. Five hundred iterations were carried out to find confirmed probes that can predict the study groups. Using confirmed 1642 probes (genes), a random forests model was built using 10,000 decision trees and importance of probes from the model was extracted. Using the top 100 probes based on their importance, the final random model was built and multi-dimensional scaling plot was generated based on dissimilarity matrix among samples obtained from the model. The *prcomp* function, *randomForest* package and *Boruta* package in R were used for the above analysis. All the codes used for the analysis have been submitted at https://github.com/rintukutum/fdr-celiac-manuscript.

## Results

### Baseline characteristics of patients

All the participants were enrolled from the Outpatients Department of Department of Gastroenterology and Human Nutrition at AIIMS, New Delhi, India. The baseline characteristics of the study participants are listed in Table 1.

**Table 1:** Baseline characteristics of study participants

	Celiac disease	FDRs	Controls
**N**	12	12	12
**Median age (years)**	21 (17–40)	33.5 (21–42)	27 (17–42)
**Males**	7	5	10
**HLADQ2/ DQ8 positive**	12	12	6

### General features of gene expression patterns in patients with CeD, FDR and controls

**Fig. 1:**
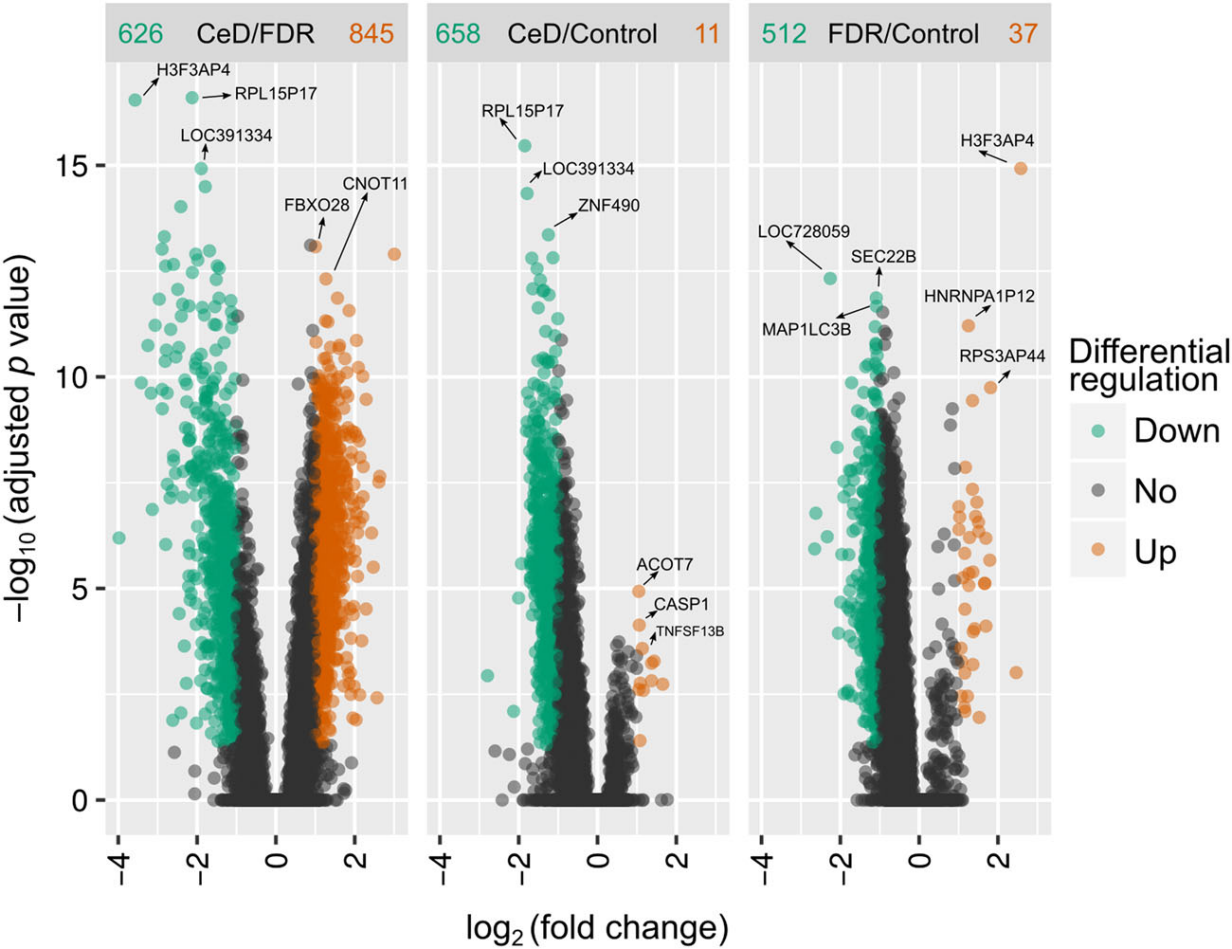
Differential gene expression patterns in the three study groups CeD, FDR and controls (*n* = 12 in each) indicate distinct transcriptomic profiles. CeD/ FDR, CeD/ Control and FDR/ Control indicate pairwise comparisons for differential expression analysis. Solid red circles indicate upregulated genes, solid green circles indicate downregulated genes, solid black/grey circles indicate no differential expression and black arrows indicate the most differentially expressed genes in each pairwise comparison

**Fig. 2:**
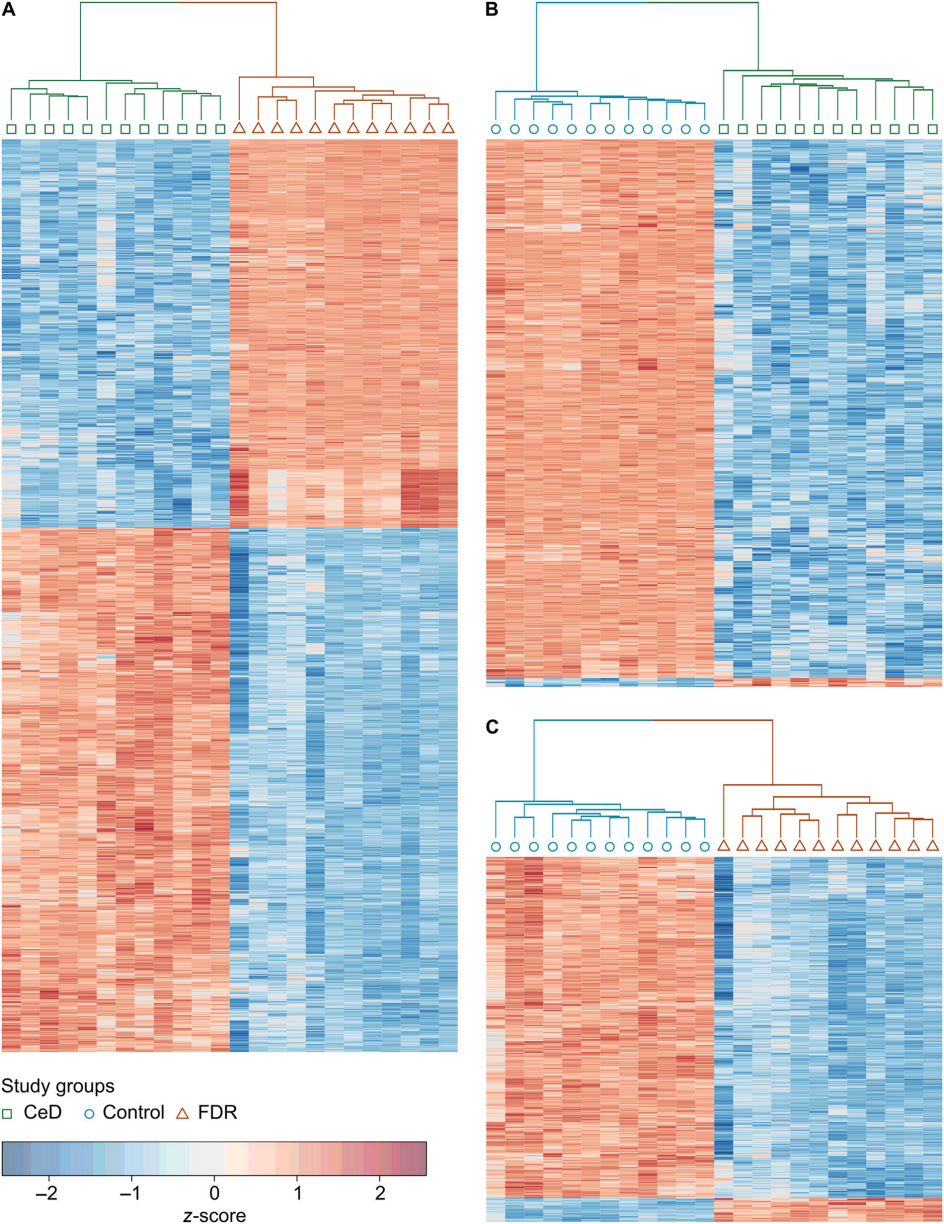
Heat map of pairwise comparisons between the three study groups CeD, FDR, and controls (*n* = 12 in each). CeD (green square), FDR (red triangle) and control (blue circle) groups have distinct gene expression patterns and hierarchical clustering groups them into distinct cohorts. There is a downregulation of most functions in the intestinal mucosa of CeD as well as FDR with respect to controls. However intestinal mucosa of FDR upregulate a specific subset of genes based on their unique functions. **A.** Pairwise analysis of FDR and CeD; ** B.** Pairwise analysis of CeD and control; ** C.** Pairwise analysis of FDR and control

Differential gene expression values obtained from pairwise analysis of the three study groups clearly show the presence of distinct significantly differentially expressed genes in each comparison (Fig. 1). The complete list of differentially regulated genes is present in Supplementary Table S1. In the comparison between CeD vs. FDR, 1471 probes were found to be differentially expressed of which 845 and 626 probes were upregulated and downregulated in CeD, respectively (Figs. 1, 2A). On the other hand, only 11 probes were found to be upregulated and 658 probes were downregulated in CeD in comparison to controls (Fig. 1, 2B). Similarly, in FDR vs. control, only 37 probes were upregulated and 512 probes were found to be downregulated in FDR (Fig. 1, 2C). The genes that were most significantly upregulated in FDR were *RPL15P17* (encoding ribosomal protein L15 pseudogene 17), *H3F3AP4* (Histone 3), *LOC391334* (gamma 2 actin, enteric smooth muscle), *HNRNPA1P12* (heterogeneous nuclear ribonucleoprotein A1 pseudogene) and RPS3AP44 (ribosomal protein S3a pseudogene 44) (Fig. 1). *FBXO28* (F-box protein 28) and *CNOT11* (CCR4-NOT transcription complex subunit 11) were found to be downregulated in the intestinal mucosa of FDR (Fig. 1). From the heatmap (Fig. 2), it is clear that the small intestinal mucosa of patients with CeD, FDR and control individuals are phenotypically distinct in terms of their global transcriptomic profiles.

**Fig. 3:**
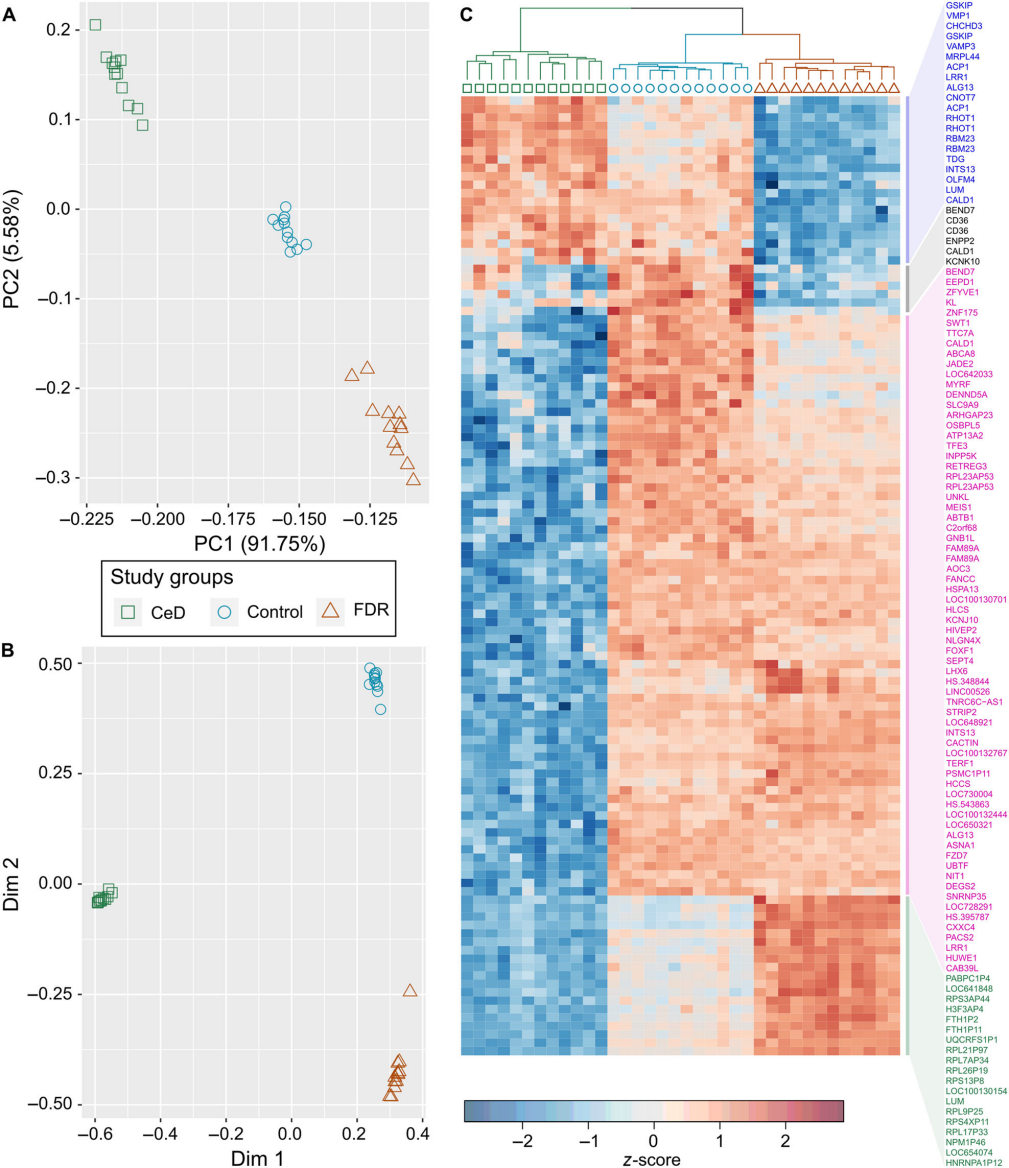
Unsupervised and supervised clustering of CeD, FDR and controls show that each group forms a distinct cluster. **A.** Unsupervised clustering using principal component analysis based on differentially expressed genes among study groups. **B.** Clustering of study groups based on random forests model with top 100 genes. **C.** Heatmap with hierarchical clustering of study groups based on top 100 genes obtained from random forests model. {CeD (green square), FDR (red triangle) and Control (blue circle)}

Unsupervised principal component analysis based on 1751 probes (genes) clearly shows segregation of CeD (green square), FDR (red triangle) and control (blue circle) groups with 96.80% variance explained (PC1 [91.75%] and PC2 [5.58%]) (Fig. 3A). FDR (red triangles) clusters further from CeD (green squares) as compared to controls (blue circles) strengthening the premise that the small intestinal mucosa of FDR have a distinct transcriptomic profile that may have active compensatory mechanisms that operate above and beyond normal intestinal mucosal functions. Based on supervised modelling using random forests and Boruta algorithm as described in methods, we could classify individuals with 100% accuracy based on top 100 probes (genes). In addition, visual inspection with multi-dimensional plot shows that the individual samples tightly clustered within their respective study groups (Fig. 3B).

Hierarchical clustering based on gene expression values of the top 100 probes (genes) that were used for final random forests model reveal that FDR, control and CeD intestinal mucosa cluster into three separate groups (Fig. 3C).

**Fig. 4:**
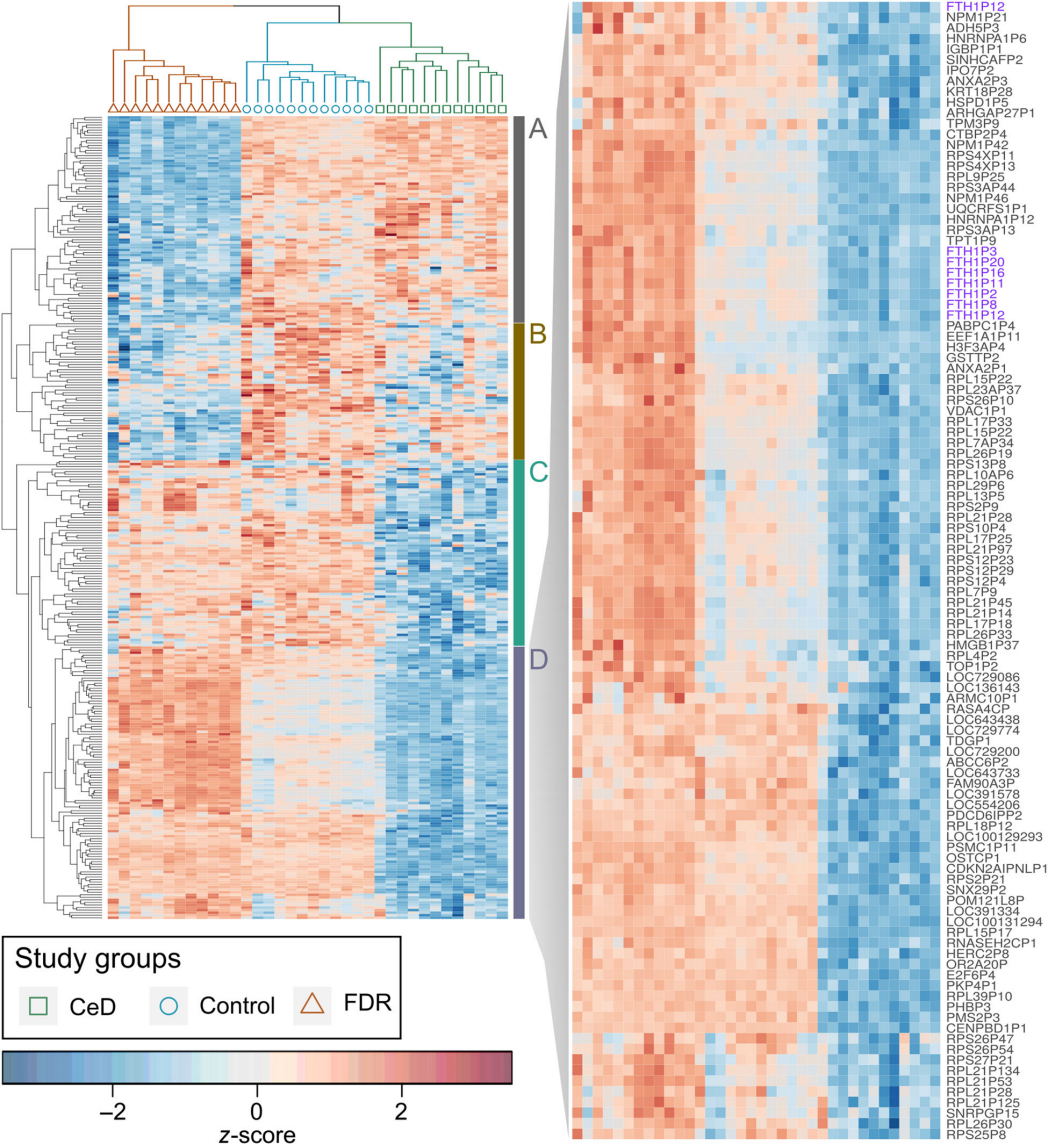
Distinct pseudogene clusters define CeD, FDR and controls. **A.** Pseudogene cluster that is down regulated in FDR but upregulated in CeD and controls. **B.** Pseudogene cluster that is downregulated in FDR, upregulated in controls but has mixed outcomes in CeD. **C.** Pseudogene cluster that is upregulated in FDR and controls but downregulated in CeD. **D.** Pseudogene cluster that is upregulated in FDR. There is a subset of pseudogenes within this cluster whose upregulation is strongly associated with FDR alone (inset). {CeD (green square), FDR (red triangle) and control (blue circle)}

A novel observation in this transcriptomic dataset was the presence of signature “pseudogene clusters” that characterize the intestinal mucosa of FDR and CeD individuals (Fig. 4). About 18% (315/1751) of the differentially expressed genes were found to be pseudogenes (Supplementary Table S2). FDR intestinal mucosa was found to be associated strongly with a cluster of pseudogenes that were specifically differentially expressed in the other two study groups of CeD and controls (Fig. 4, clusters A and D, Supplementary Figure 1A). Similarly, a specific cluster of pseudogenes was found to be associated with only CeD intestinal mucosa and was differentially expressed in FDR and Controls (Fig. 4, cluster C; Supplementary Figure 1C), whereas another different subgroup of the pseudogene cluster has a more heterogeneous expression profile across the three groups (Fig. 4, cluster B; Supplementary Figure 1B). The subgroup of pseudogenes that was found to be strongly upregulated in FDR, consisted mainly of ribosomal pseudogenes (RPS4XP11, RPS4XP13, RPL9P25, RPS3AP44, RPS3AP13), ferritin pseudogenes (FTH1P3, FTH1P20, FTH1P16, FTH1P11, FTH1P2, FTH1P8, FTH1P12), nucleophosmin pseudogenes (NPM1P42 and NPM1P46) and a few others that may be involved in the regulation of translation, cell proliferation or cell death (CTBP2P4, UQCRFS1P1, TPT1P9, GSTTP2, EEF1A1P1, ANXA2P1, PABPC1P4) (Fig. 4, cluster D).

### Validation of microarray results by qPCR

**Fig. 5:**
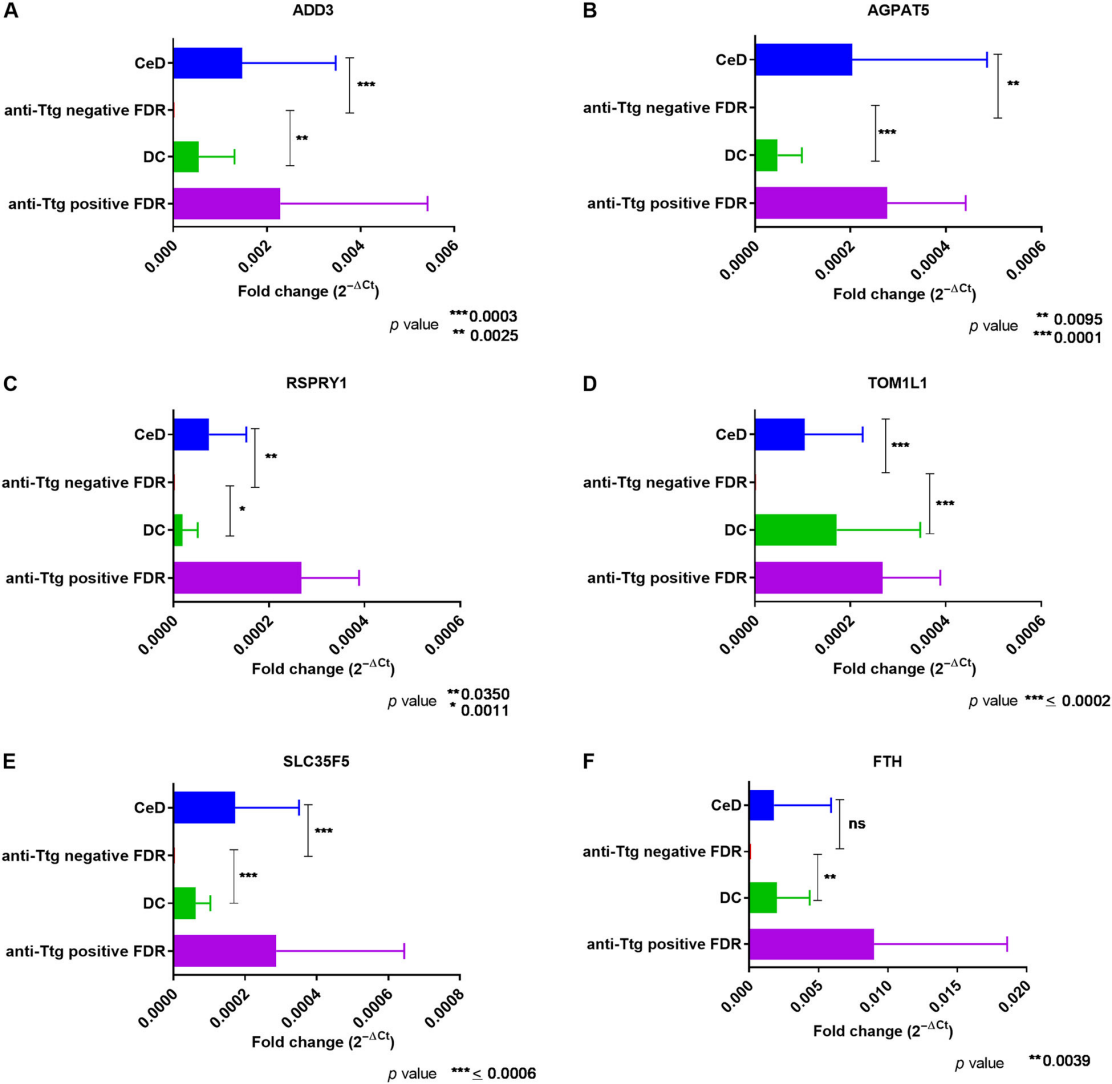
Validation of microarray data by qPCR for selected genes. In the bar graph above, the fold change (2^−ΔCT^) with standard deviation is shown for six different genes in the four study groups CeD (*n* = 8), anti-tTG negative FDR (*n* = 10), DC (*n* = 6), anti-tTG positive FDR (*n* = 2). The genes are **A.** *ADD3* (Adducin3), **B.** *AGPAT5* (1-Acylglycerol-3-Phosphate O-Acyltransferase 5), **C.** *RSPRY1* (Ring Finger And SPRY Domain Containing 1), **D.** *TOM1L1*(Target Of Myb1 Like 1 Membrane Trafficking Protein), **E.** *SLC35F5* (Solute Carrier Family 35 Member F5) and **F.**  *FTH* (Ferritin Heavy Chain 1; amplification of region common to all FTH pseudogenes)

Validation of the microarray results was carried out by qPCR analysis of duodenal biopsies obtained from consenting freshly recruited participants in the four study groups CeD (*n* = 8), anti-tTG negative FDR (*n* = 10), anti-tTG positive FDR (*n* = 2) and disease controls (DC) (*n* = 6). Therefore the validation cohort consisted of an independent group unrelated to the microarray cohort.  A set of six genes was selected for validation by qPCR, namely *ADD3* (Adducin3), *AGPAT5* (1-Acylglycerol-3-Phosphate O-Acyltransferase 5) *RSPRY1* (Ring Finger And SPRY Domain Containing 1), *TOM1L1* (Target Of Myb1 Like 1 Membrane Trafficking Protein), *SLC35F5* (Solute Carrier Family 35 Member F5) and *FTH* (Ferritin Heavy Chain 1 pseudogenes). The primers of *FTH* were designed to amplify transcripts of *FTH* pseudogenes, irrespective of type in order to quantify the total transcript levels of *FTH* pseudogenes in the FDR intestinal mucosa. The other five genes were downregulated in FDR and upregulated in both CeD and controls by microarray (Supplementary Table S1). The fold change (2^−ΔCT^) with standard deviation was calculated (Supplementary Table S3; Fig. 5). The overall gene expression patterns across the study groups in the qPCR validation were found to be similar to that in the microarray experiment. In fact, most genes downregulated in the microarray dataset were almost undetectable by qPCR in majority of the samples in the FDR group, whereas reference genes could be detected at comparable levels. Interestingly, two of the samples from the FDR group consistently showed upregulation of all the above genes resembling the gene expression pattern in CeD intestinal mucosa rather than the FDR intestinal mucosa. Upon further analysis, both were found to be anti-tTg Ab positive and segregated into a separate group designated as “anti-tTG positive FDR” and remaining samples were designated as “anti-tTG negative FDR” (Fig. 5).

### Differentially expressed pathways associated with the intestinal mucosa of FDR, CeD and controls

In order to determine the identity of differentially regulated genes that were ‘consistently’ upregulated (or downregulated) in FDR, the intersection between the genes upregulated (or downregulated) in FDR vs. control and FDR vs. CeD were considered for further analysis. Similarly, for the genes that are consistently upregulated (or downregulated) in patients with CeD, the intersection between the genes upregulated (or downregulated) in CeD vs. control and CeD vs. FDR were considered for further analysis (Supplementary Figure 2, Supplementary Table 4). This ensures that the genes analyzed are those that are consistently differentially expressed in FDR irrespective of the reference used. All the 37 genes, that were upregulated in FDR in comparison to control, were upregulated in FDR in comparison to CeD also; whereas 372 genes were found to be downregulated in FDR. (Supplementary Figure S2). In the intestinal mucosal biopsies of patients with CeD, 7 genes were upregulated (*HLA-DRB4, STAT1, TFRC, TGM2, TNFSF13B* and *ACOT7*) and 497 genes were found to be downregulated in the intersection set of CeD vs. FDR and CeD vs. controls (Supplementary Figure 2C, D).

#### (A) “Upregulated genes in the small intestinal mucosa of FDR”

Analysis of molecular function (MF) suggests that overall, the small intestinal mucosa of FDR control several pathways such as the normal metabolic functions of the enterocyte by transcriptional suppression and upregulate only a small number of pathways (Supplementary Table 5A). FDR intestinal mucosa appears to have upregulation of signatures of brush border epithelial components, as expected (Supplementary Table S5A) and transcription factors Yin Yang 1 (*YY1*), Kruppel-Like Factor 5 (*KLF5*) and *NF-ĸβ* pathway components (Bonferroni *p* value < 0.05; Supplementary Table S6).

### *(B) “Downregulated genes in the small intestinal mucosa of FDR”*

The 497 genes downregulated in FDR intestinal mucosa were subjected to pathways analysis by MSigDB and Reactome. MSigDB analysis for presence of hallmark gene signatures suggested the dowregulation of transcriptomic signatures of glycolysis, oxidative phosphorylation, fatty acid metabolism, adipogenesis and heme metabolism in the FDR intestinal mucosa (Supplementary Table S5B). Gene expression analysis revealed transcriptional suppression of genes involved in the mTOR C1 pathway and targets of c myc (Supplementary Table S5B). Reactome analysis suggested the upregulation of the β-catenin signalling pathway, which is central to the maintenance of intestinal homeostasis, since the pathway for phosphorylation of β-catenin, which leads to β-catenin degradation, was found to be downregulated in FDR intestinal mucosa (Supplementary Table S5C, yellow highlight)^13^. Reactome analysis also revealed that the other strongly downregulated processes in FDR included cross presentation of soluble exogenous antigen; (*p* = 0.007), and antigen presentation by MHC class I and II molecules (*p* = 0.007549 and 0.0108, respectively) (Supplementary Table S5C, yellow highlight).

**Fig. 6:**
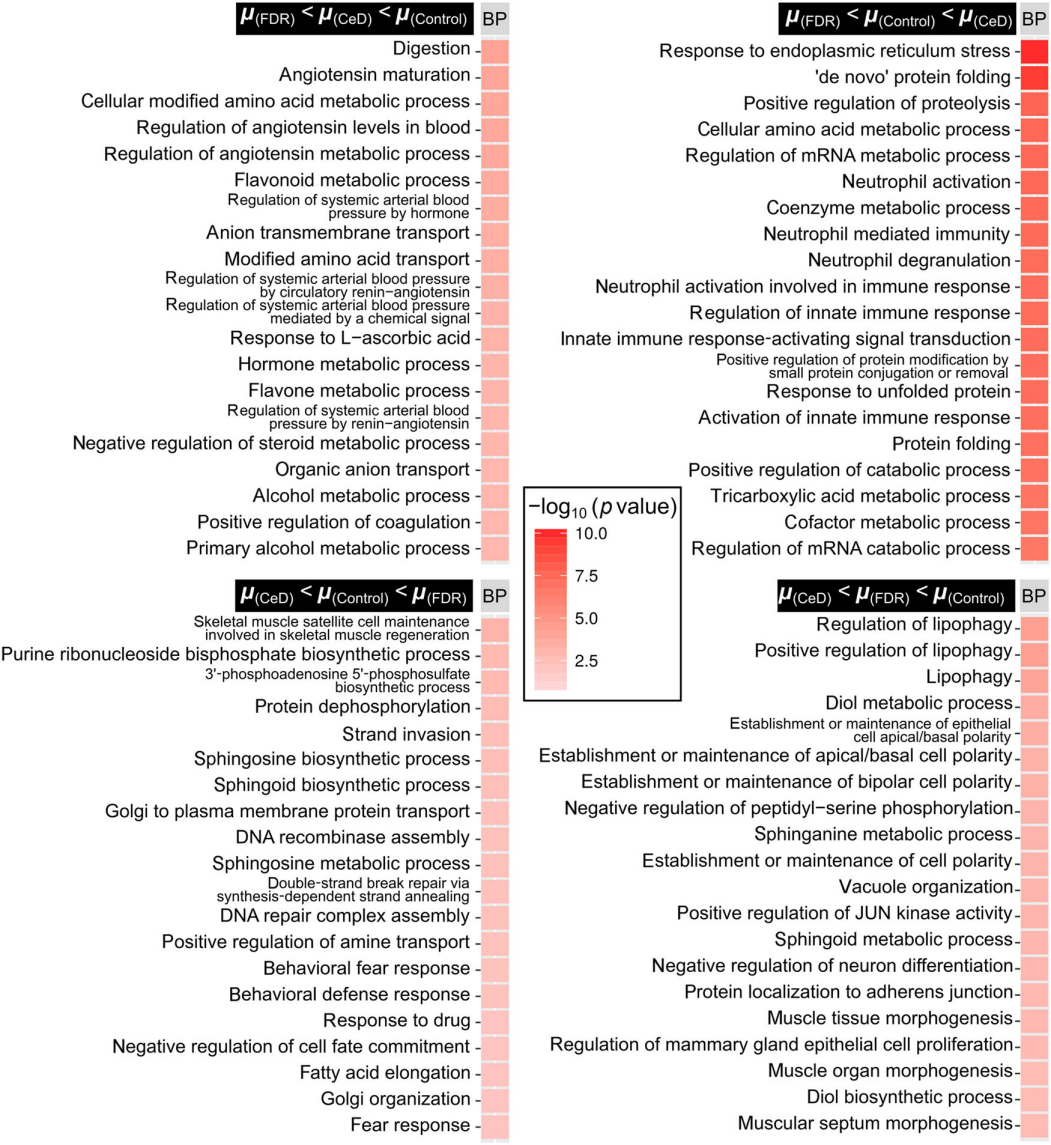
Biological processes enrichment based on pattern analysis shows that distinct processes are functional in CeD, FDR and controls. The top 20 biological processes enriched in each comparison are shown in each panel. The first quadrant (µ_FDR_ < µ_CeD_ < µ_Control_) represents the biological processes enriched for genes whose mean expression is minimum in FDR and maximum in Controls. The second quadrant represents µ_FDR_ < µ_Control_ < µ_CeD;_for processes that are minimum in FDR and maximum in CeD. The third quadrant represents µ_CeD_ < µ_FDR_ < µ_Control,_ for processes that are minimum in CeD and maximum in Controls; and the fourth quadrant represents µ_CeD_ < µ_Control_ < µ_FDR_ for processes that are minimum in CeD and maximum in FDR

The pattern analysis performed (methods; Fig. 3, Supplementary Table S7A) revealed that several key biological processes, particularly metabolic pathways, were downregulated in FDR mucosa as compared to both CeD and controls (µ_FDR_ < µ_control_ < µ_CeD_; µ_FDR_ < µ_CeD_ < µ_control_; µ_CeD_ < µ_FDR_ < µ_control_; Fig. 6, Supplementary Figures S4A and S4B). The GO biological process analysis indicated that the process of digestion was downregulated in both CeD and FDR intestinal mucosa, suggesting that some processes were similarly affected in both CeD and FDR, an indication of underlying cellular dysfunction in FDR (Fig. 6; µ_FDR_ < µ_CeD_ < µ_Control;_ Supplementary Table S7B). The process of “ER response to stress” appeared to be higher in CeD individuals as compared with FDR and controls (Fig. 6; µ_FDR_ < µ_Control_ < µ_CeD_; Supplementary Table S7B).

## Discussion

The primary consequence of gluten-induced inflammation in CeD is enteropathy, which leads to all its complications. The FDR of patients with CeD harbour the predisposing HLA-DQ2/DQ8 haplotype. While a proportion, approximately 7.5%, of FDR develop CeD, several FDR do not develop enteropathy despite having high genetic as well as environmental susceptibility for CeD. Therefore, we hypothesized the existence of protective mechanisms that may actively prevent enteropathy in FDR of patients with CeD. In order to understand the molecular phenotype of the small intestinal mucosa of FDR, we have analyzed the transcriptomic differences in the intestinal mucosa of FDR and patients with CeD and controls. Although several studies have analyzed the gene expression profiles in the intestinal mucosa of patients with CeD, this is the first study that examines the intestinal mucosal transcriptome of FDR.

The transcriptomic differences observed between the different study groups were found to be significant as judged by Bonferroni corrected *p* values and stringent fold change criteria i.e., the differences were consistent across the samples analyzed in the three study groups.

The crypt-villous axis is characterized by its tight regulation of signalling pathways and cellular processes. Our transcriptomic analysis suggests that the regulation of the crypt-villous homeostasis is the key feature that distinguishes the intestinal mucosa of FDR from CeD and control groups. Almost all the analyses performed on differentially expressed genes in FDR intestinal mucosa were found to converge at supporting robust villous growth, and the appropriate maintenance of intestinal stem cells, paneth cells and goblet cells within the crypt.

An analysis of transcription factors uncovered the upregulation of three transcription factors in the small intestinal mucosa of FDR that may have relevance in the homeostasis of the crypt-villous axis. Transcription factors such as Yin Yang 1 (*YY1*), Kruppel-Like Factor 5 (*KLF5*) and *NF-ĸβ* pathway components were found to be differentially expressed in FDR. Interestingly, two of these, *YY1* and *KLF5* are well known markers of the intestinal epithelium, and are essential for maintenance of the crypt-villous architecture and intestinal barrier function^14^. *YY1* has been shown to be important for the self-renewal of intestinal stem cells and inhibition of *YY1* causes hyper-proliferation of crypt cells without intestinal stem cell^15^. *KLF5* carries out the essential role of maintaining epithelial cell proliferation, differentiation and positioning along the crypt-villous axis. The upregulation of two Kruppel-Like factors, *YY1* and *KLF5* in the small intestinal mucosa of FDR might play a significant role in the maintenance of the crypt-villous axis in spite of low levels of enterocyte damage that might be continuing in the background. In addition, Reactome analysis shows that pathways involved in *NF-ĸβ* activation are downregulated in FDR and the inhibitory subunit *Iĸβ* is elevated in FDR, suggesting regulation of *NF-ĸβ* levels within the intestinal mucosa. Interestingly, *NF-ĸβ* has been demonstrated to be downstream to *YY1* and also has a crucial role in the maintenance of tissue homeostasis particularly at the epithelial surfaces in addition to controlling inflammation at the small intestinal mucosa^15,16^. *NF-ĸβ* is required to be present at an appropriate level, and needs to be activated to an appropriate degree for the maintenance of epithelial surfaces, including the intestinal enterocytes^17^. Activation beyond this threshold range of *NF-ĸβ* activation may lead to various inflammatory diseases at epithelial interfaces, as demonstrated in the pathogenesis of psoriasis and inflammatory bowel disease. Therefore, in the intestinal mucosa of FDR, regulation of the levels of *NF-ĸβ* might be a strategy involved in cellular regulation of the intestinal enterocytes. It is important to note however that *NF-ĸβ* signalling is modulated extensively by phosphorylation which is a post translational modification and therefore, regulation of the *NF-ĸβ* pathways may occur at multiple levels- transcriptional as well as post-transcriptional.

At the molecular level the *mTOR* (mechanistic target of rapamycin) pathway, which has been shown to be central to the maintenance of the crypt-villous axis, is regulated in FDR in a way that promotes the formation of villi. The *mTOR C1* and *c myc*- *APC* pathways have been shown to be essential in regulation of the crypt-villous architecture^18,19^. These studies also show that the *mTOR C1* pathway is progressively downregulated from the crypt to the villous and that this may be involved in regulating epithelial cell renewal along the crypt-villous axis. The disruption of genes involved in the *mTOR* pathway has been shown to improve cellular proliferation in crypts and to enhance villous growth^19^. Therefore, the regulation of the *mTOR C1* pathway may be central to the maintenance of the crypt-villous architecture in the intestinal mucosa of FDR. Similarly, a decrease in *c-myc* mediated pathways has been observed to coincide with the maturation process along the villous in gene expression studies along the crypt villous^20^. Regulation of these pathways therefore suggests an emphasis in maintaining the villous structure in the intestinal mucosa of FDR, as compared to the intestinal mucosa of CeD where villi are subjected to immune mediated enteropathy.

Transforming growth factor β (*TGF-β*), along with *Wnt*, *Notch*, *Hippo* and *Hedgehog* signalling pathways, implicated in the regulation of the crypt-villous architecture, have been reported to be under tight regulation in the small intestinal mucosa of the FDR^21^. In fact, high *TGF-β* expression is a marker for differentiated enterocytes^22^. Activation of *TGF-β* mediated pathways have been shown to aid in *β-catenin* mediated enterocyte proliferation and is associated with mucosal recovery from injury in rats^23^. Therefore, a down regulation of *TGF-β* in the intestinal mucosa of FDR, as seen in this study, suggests regulation at the level of the *TGF-β* pathway and a tight control of rapid but uncontrolled enterocyte proliferation. The transcriptomic profile of FDR suggests regulation around the *β-catenin* pathway, maintaining a balance between activation and suppression. Complete disruption of the *Wnt*/ *β-catenin* signalling pathway has been shown to cause terminal differentiation of intestinal stem cells into enterocytes. An incomplete suppression of the pathway, such as observed in the small intestinal mucosa of the FDR in the present study, might promote enterocyte differentiation of the intestinal stem cells (iSc) while still allowing maintenance of iSc population in the intestinal crypts.

Along with this intricate regulation of cellular proliferation and differentiation along the crypt-villous axis, the genes that are related to normal enterocyte metabolic functions were found to be suppressed in intestinal mucosa of FDR. This suggests that the FDR small intestinal mucosa, although apparently normal in terms of its appearance and crypt-villous ratio in histopathological analysis, is in a functionally compromised state when compared to the small intestinal mucosa of the control group. Reactome analysis revealed that immune-related functions such as antigen presentation, and activation of immune responses were downregulated in the intestinal mucosa of FDR. Therefore, the small intestinal mucosa of FDR seems to be characterized by mechanisms that actively preserve the crypt-villous axis and suppress immune responses.

A novel finding in the present study is the differential expression of “pseudogene clusters” associated with the different study groups. A large subset of pseudogenes was downregulated in FDR while the same subset was upregulated in both CeD and controls. Similarly, a distinct subset of pseudogenes was highly upregulated in FDR, giving them a distinct pseudogene signature. This suggests an important role for post transcriptional regulation mechanisms in the homeostasis of the small intestinal mucosa, particularly in the physiology of patients with CeD and their FDR. Recent research shows that a few human pseudogenes transcribe protein products and amongst the majority that do not, several regulate transcription and translation of target genes^24^. In the present study, ribosomal and ferritin pseudogenes, along with others that may have regulatory roles in cell proliferation and death, were found to be highly upregulated in the intestinal mucosa of FDR. Ribosomal pseudogene expression has been shown to be quite abundant in various tissues and they constitute about 20% of all human processed pseudogenes^25^. Among the ferritin pseudogenes, *FTH1P3* has been shown to be transcriptionally active in several different cell types and tissues^26^. *FTH1P3* is a low copy number transcript in comparison to the parent gene encoding for Ferritin Heavy Chain (*FHC*) and the regulation of *FTH1P3* and *FHC* transcription appear to be independent of each other, suggesting that upregulation of *FTH1P3* might have a ferritin-independent regulatory role in tissues. In the colon carcinoma Caco-2 cell line, which spontaneously differentiate into enterocyte-like cells in culture, *FTH1P3* levels have been shown to increase threefold in fully differentiated enterocyte-like cells as compared to undifferentiated cells implying that *FTH1P3* might be a marker for enterocyte differentiation^27^.

The significance of these pathways in maintaining intestinal homeostasis requires further experimental validation. However, this study provides an essential first glimpse into the different processes that interact within the intestinal mucosa to maintain structure and function of the crypt-villous axis, in spite of genetic predisposition and presence of environmental triggers for immune-mediated cellular damage. A limitation of our study is the absence of matched CeD-FDR pairs which would have minimized host variability in the study. The limitation is due to the inclusion of only those participants who provided informed consent to be a part of the study. However, an advantage of the above approach is the random inclusion of CeD and unmatched FDR from the general population, thereby minimizing any bias.

In conclusion, our study suggests that the intestinal mucosa of FDR consist of a unique molecular phenotype that is distinct from CeD patients and controls. The transcriptomic landscape of FDR promotes maintenance of crypt-villous axis, as well as modulation of immune responses while suppressing other pathways that are functional in normal intestinal mucosa as represented by the control group. These differences clearly show the existence of compensatory mechanisms that may protect FDR from developing enteropathy.

## Study Highlights

### What is current knowledge?

- Celiac disease (CeD) has genetic associations with both- HLA and non-HLA genes.
- About 7.5% of first-degree relatives of patients with celiac disease (FDR) are at a higher risk of developing the disease; many however, are protected from developing the enteropathy related to celiac disease
- Protective mechanisms that prevent development of enteropathy in the FDR of patients with celiac disease are unknown.

### What is new here?

- The molecular phenotype of small intestinal tissue from the FDR of CeD patients has been investigated for the first time.
- The intestinal mucosa of FDR, harbour a combination of pathways that focus on maintenance of the crypt-villous architecture, and on regulation of immune homeostasis within the small intestine.
- We find association of the FDR intestinal mucosa with novel and unique “pseudogene clusters” that may be involved in transcriptomic regulation.
- These mechanisms may help the FDR small intestinal mucosa maintain its structure and prevent enteropathy despite genetic predisposition and presence of environmental trigger to develop immune mediated cellular damage in the small intestine.

## Electronic supplementary material

Supplementary Figure

Supplementary Figure

Supplementary Figure

Supplementary Figure

Supplementary Figure

Supplementary Notes

Supplementary Information
