# Supplementary figures and images for: First Degree Relatives of Patients with Celiac Disease Harbour an Intestinal Transcriptomic Signature that Might Protect them from Enterocyte Damage
Source: Clin Transl Gastroenterol. 2018 Oct 8;9(10):195. doi: 10.1038/s41424-018-0059-7 (PMC6174158; doi:10.1038/s41424-018-0059-7)

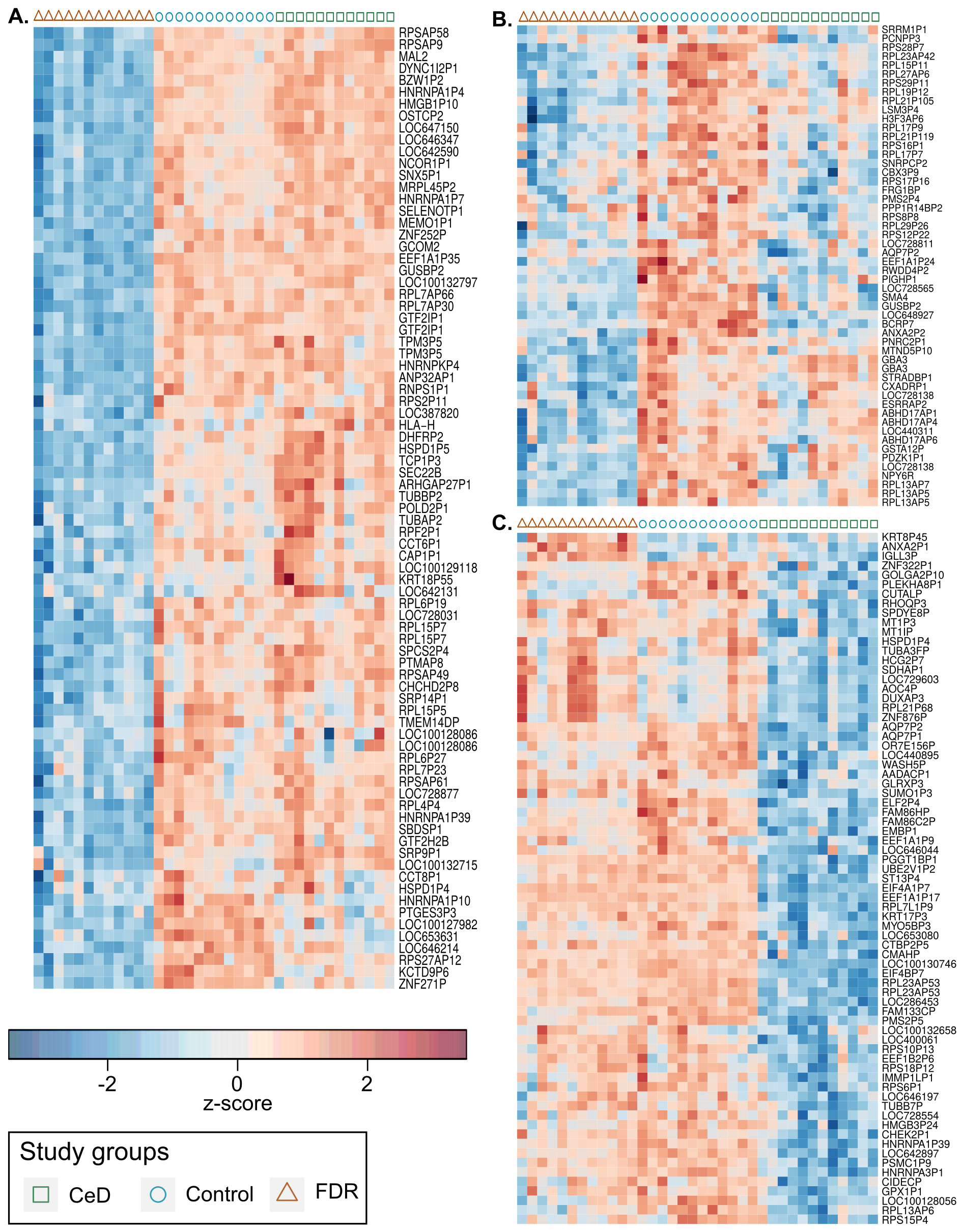

Supplement: Supplementary file 1 — Supplementary Figure [file 41424_2018_59_MOESM1_ESM.tiff]

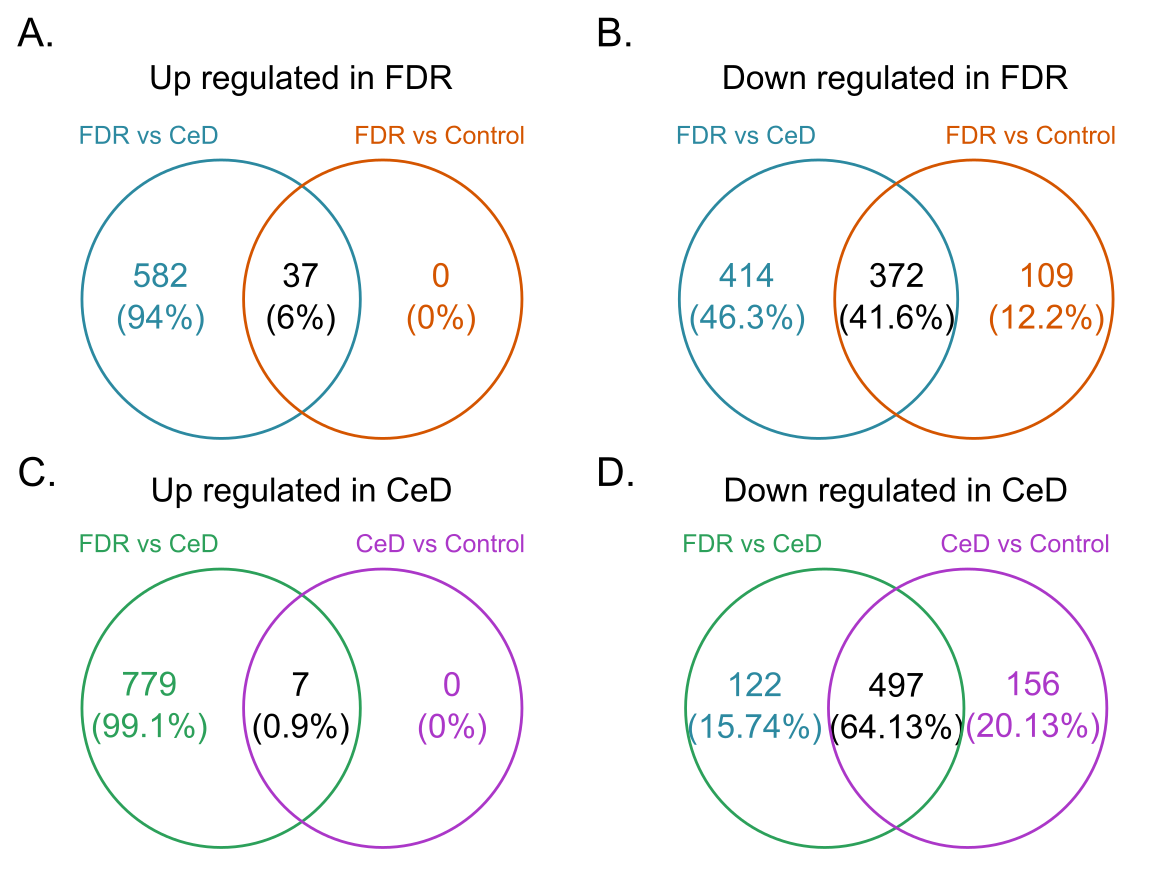

Supplement: Supplementary file 2 — Supplementary Figure [file 41424_2018_59_MOESM2_ESM.tiff]

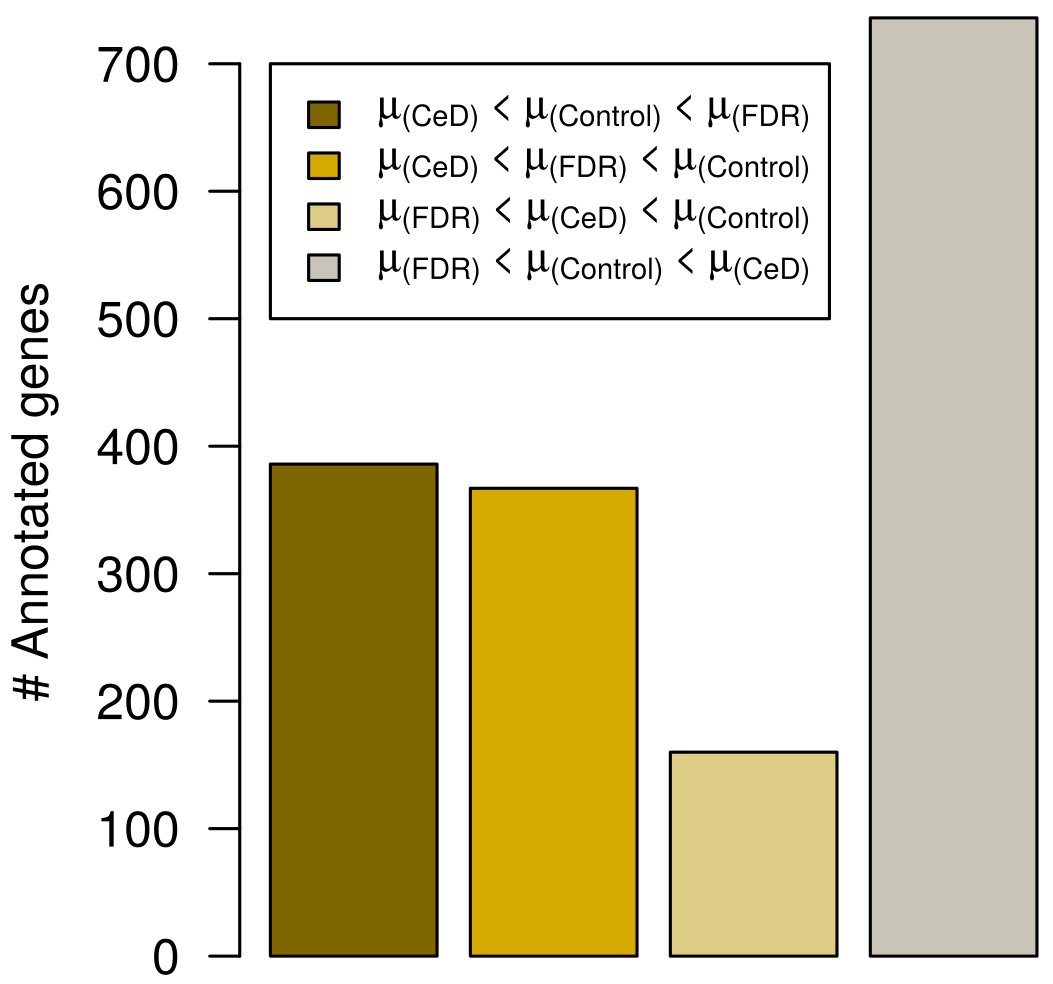

Supplement: Supplementary file 3 — Supplementary Figure [file 41424_2018_59_MOESM3_ESM.tiff]

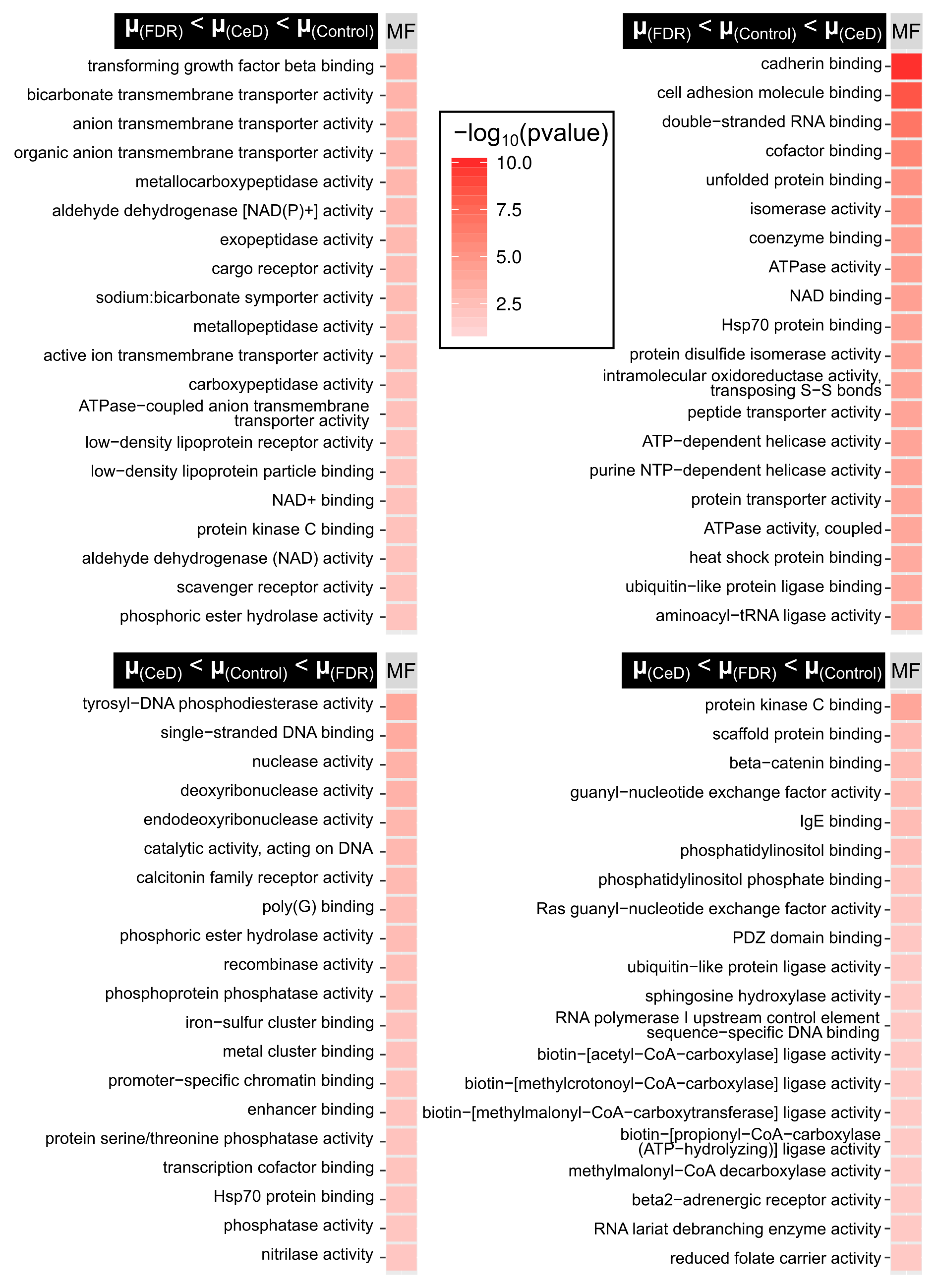

Supplement: Supplementary file 4 — Supplementary Figure [file 41424_2018_59_MOESM4_ESM.tiff]

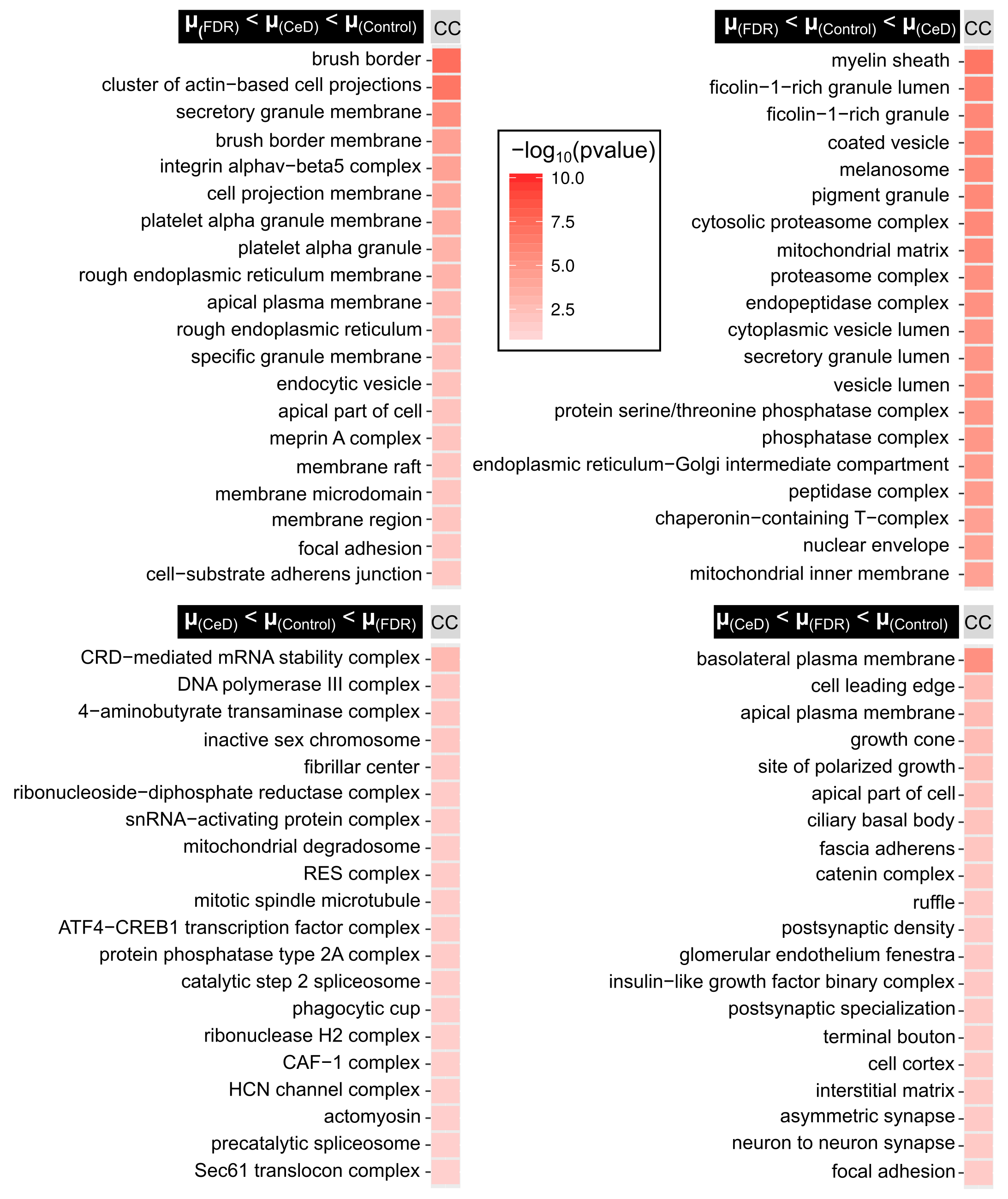

Supplement: Supplementary file 5 — Supplementary Figure [file 41424_2018_59_MOESM5_ESM.tiff]
