# Supplementary material for: First Degree Relatives of Patients with Celiac Disease Harbour an Intestinal Transcriptomic Signature that Might Protect them from Enterocyte Damage
Source: Clin Transl Gastroenterol. 2018 Oct 8;9(10):195. doi: 10.1038/s41424-018-0059-7 (PMC6174158; doi:10.1038/s41424-018-0059-7)
Supplement: Supplementary file 6 — Supplementary Notes [file 41424_2018_59_MOESM6_ESM.docx]

**Supplementary Notes:**

**This file contains the location of ALL the figures, tables and supplementary material used to reach the conclusions in the manuscript. It is freely accessible to everyone using the links given below.**

All the R scripts used for the analysis and figure generation were deposited at github (<https://github.com/rintukutum/fdr-celiac-manuscript>) and finally the final version have been archive at zenodo (<https://doi.org/10.5281/zenodo.1326188>)

Composite figures were generated using inkscape and corresponding .svg files have been also uploaded in the github repository. (<https://github.com/rintukutum/fdr-celiac-manuscript/tree/master/figures/svg>)

**Figures for manuscript (**<https://github.com/rintukutum/fdr-celiac-manuscript/tree/master/figures/tiff>**):**

**Figure1:** Volcano plots

**Figure2:**Heatmaps

**Figure3:** PCA, RF and Heatmap

**Figure4:** Pseudogenes expression profile; The cluster D with the ferretin genes are highlighted with light purple color.

**Figure5:** qPCR

**Figure6:** GO enrichment for top 10 biological processes across gene expression patterns.

**Figure-S1:** Pseudogenes cluster A, B and C.

**Figure-S2:** Venn-diagram of up and down regulated genes in FDR and CeD.

**Figure-S3:** Overall pattern analysis stats

**Figure-S4:** a) pattern analysis MF and b) pattern analysis CC

**Supplementary Tables**

All the supplementary tables (excel and pdf) were uploaded at zenodo ([https://zenodo.org](https://zenodo.org/)).

**1. Table-S1-Differentially-expressed-genes.xlsx**

List of differentially expressed probes(genes) based on ajusted-pvalue<= 0.05 and |log2(FC)| >= 1. <http://doi.org/10.5281/zenodo.1296053>

**2. Table-S2-pseudogenes-DE-clsuter-ID-annotations.xls**

List of pseudogenes differentially expressed among CeD, FDR and Control.

<https://doi.org/10.5281/zenodo.1322802>

**3. Table-S3_qPCR_gene_expression_fold_change.pdf**

Gene expression fold-change of study groups: Anti-tTG positive FDR, CeD, DC, Anti-tTG negative FDR. <http://doi.org/10.5281/zenodo.1322812>

**4. Table-S4-data-for-FigureS2-venn-diagram.xlsx**

List of genes up-regulated and down-regulated in FDR and CeD. <http://doi.org/10.5281/zenodo.1322817>

**5a. Table-S5a-GO-pval-0.05-DE.xlsx**

Gene ontology enrichment results for differentially expressed genes among FDR, CeD and Control.

<http://doi.org/10.5281/zenodo.1322806>

**5b. Table-S5b-MsigDB_Consistently_down_in_FDR.xlsx**

MSigDB enrichment analysis based on genes which are down-regualted consistently in FDR.

<http://doi.org/10.5281/zenodo.1322807>

**5c. Table-S5c-Reactome-Down-in-FDR.xlsx**

Reactome pathway enrichment for down in FDR based on adjusted-pvalue<= 0.05 and without log2(FC) criteria.

<https://doi.org/10.5281/zenodo.1324239>

**6. Table-S6-TranscriptionFactors-YY1-KLFs-NFKB-inhibitors.xlsx**

Detail information of p-value, log2(FC), adjusted-pvalue etc. for selected tanscription factors.

<http://doi.org/10.5281/zenodo.1322816>

**7a. Table-S7a-pattern-analysis.xlsx**

List of probes/genes with their gene expression pattern among CeD, FDR and Control.

<http://doi.org/10.5281/zenodo.1322814>

**7b. Table-S7b-GO_pattern.xlsx**

Gene ontology enrichment analysis based on genes for each expression pattern observed among CeD, FDR and Control. <http://doi.org/10.5281/zenodo.1322813>
