# Supplementary material for: First Degree Relatives of Patients with Celiac Disease Harbour an Intestinal Transcriptomic Signature that Might Protect them from Enterocyte Damage
Source: Clin Transl Gastroenterol. 2018 Oct 8;9(10):195. doi: 10.1038/s41424-018-0059-7 (PMC6174158; doi:10.1038/s41424-018-0059-7)
Supplement: Supplementary file 7 — Supplementary Information [file 41424_2018_59_MOESM7_ESM.dotx]

**qPCR information in accordance to MIQE^1^ guidelines**

**I. Experimental design:**

(i) Study groups:

- First degree relative individuals, tested negative for anti-tTG antibody ( anti- tTG negative FDR, n = 10 )
- Celiac Disease patients (CeD, n = 8 )
- Disease controls (e.g. GERD, Dyspepsia) (n = 6)
- First degree relative individuals, tested positive for anti- tTG antibody ( anti- tTG positive FDR, n = 2)

**II. Sample:**

1. **Volume/mass of sample processed:** 50-100 mg of duodenal tissue sample obtained via biopsy during endoscopy of the second duodenal region, was collected in 500 µl of RNA later (Cat. No: G46481A; GCC BIOTECH) after written informed consent. The samples were transferred to 1 ml of TRI REAGENT (Cat No. TR 118) and processed according to the standardized laboratory protocol.
2. **Total RNA extraction :** Tissue samples were processed for RNA isolation by conventional method using TRIzol® (Thermofisher Scientific), as follows: Approximately 50-100mg of intestinal tissue sample was transferred to 1ml of Trizol. The tissue was homogenized using a sterile Teflon homogenizer and incubated at room temperature for 5-10 minutes, with intermittent vortexing. To the homogenized samples, 0.2ml of Chloroform was added, and the samples were mixed and vortexed vigorously, followed by centrifugation at 12,000 X g for 15 minutes, at 4°C. The resulting aqueous upper phase was transferred to a sterile micro-centrifuge tube, and 0.5 ml of Isopropanol was added. The samples were kept overnight at -20°C for RNA precipitation. The following day, the samples were centrifuged at 12,000 X g for 15 minutes, at 4°C. The supernatant was discarded and RNA pellet was washed with 75% ethanol once, and air dried at 37°C for 10 minutes. The dried pellet was resuspended in 30µl of  RNase-free water. The RNA was quantified using NanoDrop™ 2000/2000c Spectrophotometer- Thermo Fisher Scientific and RNA integrity was checked by running the RNA on 1.5% agarose gel.
3. The RNA yield (ng/µl) and A_260/280_ ratios for the experimental and control samples are as follows:

| Group | Sample I.D. | RNA yield (ng/uL) | A_260/280_ |
| --- | --- | --- | --- |
| Anti-tTG negative FDR | FDR_1 | 874.0 | 1.90 |
|  | FDR_2 | 1319.80 | 1.90 |
|  | FDR_3 | 1577.00 | 1.93 |
|  | FDR_4 | 677.20 | 2.00 |
|  | FDR_5 | 2488.60 | 1.89 |
|  | FDR_6 | 406.40 | 1.78 |
|  | FDR_7 | 614.50 | 1.8 |
|  | FDR_8 | 790.00 | 1.85 |
|  | FDR_9 | 3661.90 | 1.57 |
|  | FDR_10 | 1249.00 | 1.89 |
| Celiac Disease | CeD_1 | 2040.90 | 1.82 |
|  | CeD_2 | 1657.28 | 1.19 |
|  | CeD_3 | 1037.10 | 1.97 |
|  | CeD_4 | 314.20 | 2.24 |
|  | CeD_5 | 396.90 | 2.02 |
|  | CeD_6 | 351.30 | 2.01 |
|  | CeD_7 | 404.70 | 1.97 |
|  | CeD_8 | 306.30 | 2.14 |
| Disease Control | DC_1 | 671.78 | 0.68 |
|  | DC_2 | 267.00 | 0.29 |
|  | DC_3 | 625.31 | 0.51 |
|  | DC_4 | 466.13 | 0.29 |
|  | DC_5 | 241.90 | 2.19 |
|  | DC_6 | 176.80 | 2.41 |
| anti-tTG positive FDR | ttg_FDR_1 | 410.80 | 1.98 |
|  | ttg_FDR_2 | 584.90 | 1.89 |

1. **Reverse transcription:** Total RNA was reverse transcribed into cDNA using verso cDNA synthesis kit (AB-1453/A from Thermo Fisher Scientific).The constituents of the 20 µl reaction consisted of: Buffer = 4µl, dNTP = 2 µl, Primer (Random hexamer: anchored oligo dT = 3:1) = 1 µl, Reverse Transcriptase enzyme = 1 µl, Enhancer ( DNAse ) = 1µl. The cDNA synthesis was carried out from 2µg of RNA. The total volume was adjusted upto 20µl with Nuclease free water and the reaction was carried out at 42 °C for 30 minutes followed by 2 minutes incubation at 95°C
2. **Storage:** The cDNA was aliquoted and stored at -20°C until further use.

**III. qPCR target information**

**(i) Genes targeted in qPCR experiments are as follows:** Ring finger and SPRY domain containing 1 (RSPRY1), Target of Myb1 like 1 membrane trafficking protein (TOM1L1), Solute carrier family 35, member F5 (SLC35F5), 1-acyl glycerol-3-phosphate-O-acyltransferase-5 (AGPAT5), Adducin-3-gamma (ADD3), Ferritin Heavy chain (FTH) and 18S rRNA housekeeping gene.

**(ii) Primer designing strategy:** The primers against the set of referred genes were designed using NCBI primer BLAST software. The design parameters settings were chosen to allow primers span the exon-exon junctions and intron-exclusion criteria. Also primer designs were allowed to amplify mRNA splice variants.

The  specificity of designed primers was verified by BLAST platform of NCBI.

**(iii) Target information with NCBI gene accession number and primer sequences:**

**(iii) Manufacturer of oligonucleotides:** Eurofins pvt. Ltd. India.

**(iv) PCR standardization and primer efficiency:**

A total of 6 genes were selected for standardization: ADD3, AGPAT5, RSPRY1, TOM1L1, SLC35F5 and FTH. 18S rRNA war used as the housekeeping gene. Primer validations and standardization of the reaction conditions were done by temperature gradient PCR in Agilent SureCycler 8800. Reaction conditions were optimized to be same for all the genes, and chosen for qPCR as follows: 95°C 3’, 95°C 30’’, 60°C 20’’, 72°C 20’’, for 35 cycles.

Primer efficiency was measured for each primer pair by the standard curve method, using a 2-fold dilution series of the pooled cDNA template from each of the groups: FDR, CeD, and DC samples, by qPCR.

**IV. qPCR protocol**:

**(i) Complete reaction conditions:** SYBR green 1 chemistry was used to determine the relative expression of the target genes. The cycling parameters are: Initial denaturation at 95°C for 3 minutes, followed by annealing, amplification and detection for 35 cycles (95°C 30’’, 60°C 20’’, 72°C 20’’) at end-point fluorescence, and melt-curve analysis at 60-95°C at 0.1 °C rise per second at continuous fluorescence to detect specific amplicons.

**(ii) Reaction volume and components:** Reaction volume of total 10 ul: Components were 9 µl mastermix consisting of 5 µl of SYBR Green buffer. Mastermix (Thermo Fisher Scientific Dynamo Flash: F415S ), 0.4 µl of forward + 0.4 µl of reverse primers (0.4mM), 1.6 µl of nuclease-free water, and 30 ng of template cDNA.

**(v) Manufacture of qPCR instruments:** Bio-Rad CFX-96 well plate real-time PCR machine was used to carry out the qPCR experiments.

**V. Quantitative real-time PCR:** Attached supplementary data table S3.

**References:**

**(In accordance with the MIQE^1^ guidelines):**<http://clinchem.aaccjnls.org/content/55/4/611>
